# A dataset of continuous affect annotations and physiological signals for emotion analysis

**DOI:** 10.1038/s41597-019-0209-0

**Published:** 2019-10-09

**Authors:** Karan Sharma, Claudio Castellini, Egon L. van den Broek, Alin Albu-Schaeffer, Friedhelm Schwenker

**Affiliations:** 1 Institute of Robotics and Mechatronics, DLR–German Aerospace Center, Wessling, Germany; 2 Agile Robots AG, Gilching, Germany; 3 Department of Information and Computing Sciences, Utrecht University, Utrecht, The Netherlands; 4 Institute of Neural Information Processing, Ulm University, Ulm, Germany

**Keywords:** Electrocardiography - EKG, Electromyography - EMG, Biomedical engineering, Cognitive neuroscience, Physiology

## Abstract

From a computational viewpoint, emotions continue to be intriguingly hard to understand. In research, a direct and real-time inspection in realistic settings is not possible. Discrete, indirect, post-hoc recordings are therefore the norm. As a result, proper emotion assessment remains a problematic issue. The Continuously Annotated Signals of Emotion (CASE) dataset provides a solution as it focusses on real-time continuous annotation of emotions, as experienced by the participants, while watching various videos. For this purpose, a novel, intuitive joystick-based annotation interface was developed, that allowed for simultaneous reporting of valence and arousal, that are instead often annotated independently. In parallel, eight high quality, synchronized physiological recordings (1000 Hz, 16-bit ADC) were obtained from ECG, BVP, EMG (3x), GSR (or EDA), respiration and skin temperature sensors. The dataset consists of the physiological and annotation data from 30 participants, 15 male and 15 female, who watched several validated video-stimuli. The validity of the emotion induction, as exemplified by the annotation and physiological data, is also presented.

## Abstract (summary)

**Table:** 

Measurement(s)	electrocardiogram data • respiration trait • blood flow trait • electrodermal activity measurement • temperature • muscle electrophysiology trait
Technology Type(s)	electrocardiography • Hall effect measurement system • photoplethysmography • Galvanic Skin Response • skin temperature sensor • electromyography
Factor Type(s)	sex • age
Sample Characteristic - Organism	Homo sapiens

Machine-accessible metadata file describing the reported data: 10.6084/m9.figshare.9891446

## Background & Summary

The field of *Artificial Intelligence* (AI) has rapidly advanced in the last decade and is on the cusp of transforming several aspects of our daily existence. For example, services like customer support and patient care, that were till recently only accessible through human–human interaction, can nowadays be offered through AI-enabled conversational chatbots^1^ and robotic daily assistants^2^, respectively. These advancements in interpreting explicit human intent, while highly commendable, often overlook implicit aspects of human–human interactions and the role emotions play in them. Addressing this shortcoming is the aim of the interdisciplinary field of *Affective Computing* (AC, also known as *Emotional AI*), that focuses on developing machines capable of recognising, interpreting and adapting to human emotions^3,4^.

A major hurdle in developing these *affective* machines is the internal nature of emotions that makes them inaccessible to external systems^5^. To overcome this limitation, the standard AC processing pipeline^6^ involves: (i) acquiring measurable indicators of human emotions, (ii) acquiring subjective annotations of internal emotions, and (iii) modelling the relation between these indicators and annotations to make predictions about the emotional state of the user. For undertaking steps (i) and (ii) several different strategies are used. For example, during step (i) different modalities like physiological signals^5,7,8^, speech^9^ and facial-expressions^10,11^ can be acquired. Similarly, the approaches to step (ii) vary along the following two main aspects. First, on the kind of annotation scale employed, i.e., either discrete or continuous. Second, on the basis of the emotion-model used, i.e., either discrete emotion categories (e.g., joy, anger, etc.) or dimensional models (e.g., the *Circumplex model*^12^). Traditionally, approaches based on discrete emotional categories were commonly used. However, these approaches were insufficient for defining the intensity^6,8,11^ and accounting for the time-varying nature^13^ of emotional experiences. Therefore, continuous annotation based on dimensional models is preferred and several annotation tools for undertaking the same have been developed^14–16^. Notwithstanding these efforts at improving the annotation process, a major impediment in the AC pipeline is that steps (i) and (ii) require direct human involvement in the form of subjects from whom these indicators and annotations are acquired^8,17^. This makes undertaking these steps a fairly time-consuming and expensive exercise.

To address this issue, several (uni- and multi-modal) datasets that incorporate continuous annotation have been developed. Principal among these are the DEAP^18^, SEMAINE^19^, RECOLA^20^, DECAF^21^ and SEWA^22^. The annotation strategy used in these datasets have the following common aspects. First, the two dimensions of the Circumplex model (i.e., *valence* and *arousal*) were annotated separately. Second, in all datasets except SEWA, that uses a joystick, mouse-based annotation tools were used. In recent years, both these aspects have been reported to have major drawbacks^22–24^. These being, that separate annotation of valence and arousal does not account for the inherent relationship between them^23,25^, and that mouse-based annotation tools are generally less ergonomic than joysticks^22–24,26^. To address these drawbacks, we developed a novel *Joystick-based Emotion Reporting Interface* (JERI) that facilitates simultaneous annotation of valence and arousal^16,27–29^. A testament to the efficacy of JERI is that in recent years, several similar annotation setups have been presented^25,30^. However, currently there are no openly available datasets that feature JERI-like setups.

To address this gap, we developed the *Continuously Annotated Signals of Emotion* (CASE) dataset^31,32^. It contains data from several physiological sensors and continuous annotations of emotion. This data was acquired from 30 subjects while they watched several video-stimuli and simultaneously reported their emotional experience using JERI. The physiological measures are from the Electrocardiograph (ECG), Blood Volume Pulse (BVP), Galvanic Skin Response (GSR), Respiration (RSP), Skin Temperature (SKT) and Electromyography (EMG) sensors. The annotation data has been previously used for several publications aimed at introducing, analysing and validating this approach to emotion annotation^16,28,29^. However, it has not been previously released. To the best of our knowledge, this is the first dataset that features continuous and simultaneous annotation of valence and arousal, and as such can be useful to the wider Psychology and AC communities.

## Methods

### Participants

Thirty volunteers (15 males, age 28.6 ± 4.8 years and 15 females, age 25.7 ± 3.1 years; range of age 22–37 years) from different cultural backgrounds participated in the data collection experiment. These participants were recruited from an organisation-wide call for volunteers sent at the Institute of Robotics and Mechatronics, DLR. Upon registering for the experiment, an email containing general information and instructions for the experiment was sent to the participants. In this email, they were asked to wear loose clothing and men were asked to preferably shave facial hair, to facilitate the placement of sensors. All participants had a working proficiency in English and were communicated to in the same. More information on the sex, age-group, etc., of the participants is available in the metadata to the dataset^31,32^.

### Ethics statement

This experiment is compliant with the World Medical Association’s Declaration of Helsinki, that pertains to the ethical principles for medical research involving human subjects, last version, as approved at the 59th WMA General Assembly, Seoul, October 2008. Data collection from participants was approved by the institutional board for protection of data privacy and by the work council of the German Aerospace Center. A physician is part of the council that approved the experiment.

### Experiment design

The experiment was setup with a *within subjects design*. Accordingly, *repeated measures* were made and all participants watched and annotated the different video-stimuli used for the experiment. To avoid carry-over effects, the order of the videos in a viewing session was modified in a pseudo-random fashion, such that the resulting video sequence was different for every participant. To isolate the emotional response elicited by the different videos, they were interleaved by a two-minute long blue screen. This two-minute period also allowed the participants to rest in-between annotating the videos. More information on the video-sequences is available in the dataset^31,32^.

### Experiment protocol

**Fig. 1:**
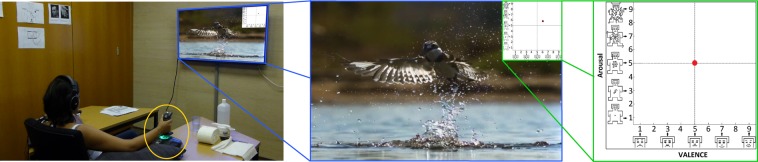
The typical experiment setup shows a participant watching a video and annotating using JERI. The central figure shows the video-playback window with the embedded annotation interface. The right-most figure shows the annotation interface in detail, where Self-Assessment Manikin that were added to the valence and arousal axes can be seen.

On the day of the experiment, the participants were provided an oral and a written description of the experiment. The written description is available as a supplementary file to this document. After all questions regarding the experiment were addressed, the participants were asked to sign the informed consent form. Then, a brief introduction to the 2D circumplex model was provided and any doubts about the same were clarified. Following this, physiological sensors were attached and the participant was seated facing a 42” flat-panel TV (see Fig. 1, left). Detailed information was then provided on the annotation procedure. It was emphasised to the participants that they should annotate their emotional experience resulting from the videos, and not the emotional content of the videos. To accustom the participants to the annotation interface, they were asked to watch and annotate five short practice videos. During this practice session, the experiment supervisor intervened whenever the participant asked for help and if required, provided suggestions at the end of every video. This session was also used to inspect the sensor measurements and if required, make appropriate corrections. After the practice session, the experiment was initiated and lasted for approximately 40 minutes. During the experiment, the supervisor sat at a computer table placed roughly two meters behind the participants to monitor the data acquistion, but not to specifically observe the annotation style of the participants. The participants were informed about the reasons of his presence. At the end of the experiment, feedback on the annotation system was acquired using the SUS questionnaire^16,33^. Then, the sensors were removed and refreshments were offered. The participants were also encouraged to share any further insights they had on the experiment.

### Annotation interface

**Fig. 2:**
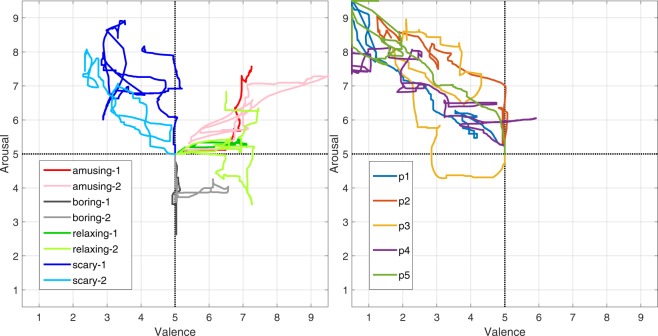
The plot on the left shows the annotations from one participant for the different videos (see Table 1) in the experiment. The annotations for the ‘scary-2’ video by the first five participants (labelled as p1–p5) can be seen in the plot on the right.

Figure 1 (right) shows the design of the annotation interface. It is based on the 2D circumplex model that has been supplemented with the Self-Assessment-Manikin (SAM)^34^ on its co-ordinate axes. These manikin depict different valence (on X–axis) and arousal (on Y–axis) levels, thereby serving as a non-verbal guide to the participants during annotation. The red pointer in the figure shows the resting/neutral position. The participants were instructed to annotate their emotional experience by moving/holding the red pointer in the appropriate region of the interface. The position of the annotation interface inside the video-playback window is shown in Fig. 1 (center). This position can be easily changed, but since none of the participants requested that, it was retained as shown for all participants. Since the annotation was done over the entire length of a video, it results in a continuous 2D trace of the participant’s emotional experience (see Fig. 2). The annotation interface was developed in the National Instruments (NI) LabVIEW programming environment.

### Videos

#### Practice videos

As previously mentioned, the aim of the practice session was to acquaint the users to the annotation interface and not to explicitly train them on the different types of emotional videos they would encounter in the main experiment. Accordingly, five short (duration: ~1 minute each) videos interleaved by a 10-second long transitioning blue screen, were used for this session. These videos aimed to elicit *exciting, sad, relaxing, scary* and *happy* emotional states. Thus, the expected emotional content of these videos was often similar to the videos used in the main experiment, but not in all cases. The data acquired during the practice session was not recorded and is therefore not a part of this dataset.

#### Experiment videos

In the main experiment, the aim was to elicit *amusing, boring, relaxing* and *scary* emotional states through video-stimuli. To this end, 20 videos previously used by other studies were shortlisted^35–37^. The emotional content of these videos was then verified in a pre-study, where 12 participants (no overlap with the participants of this study) viewed and rated these videos remotely using a web-based interface. Based on the results of this pre-study and further internal reviews, eight videos were selected for the main experiment, such that there were two videos each for the emotional state that we wanted to elicit. Additionally, three other videos were also used in the experiment, i.e., the start-video, the end-video and the interleaving blue-screen videos. The start-video is a *relaxing* documentary excerpt aimed at calming participants before the presentation of the emotional videos. The end-video was added for the same purpose, i.e., to act as a ‘cool-down’ phase before the end of the experiment. More information on all these videos is available in Table 1, in the Usage Notes section and in the dataset^31,32^.

**Table 1:** The source, label, ID used, intended valence-arousal attributes and the duration of the videos used for the dataset.

Source	Video-Label	Video-ID	Intended Attributes		Dur. [*s*]
			Valence	Arousal	
Hangover	amusing-1	1	med/high	med/high	185
When Harry Met Sally	amusing-2	2	med/high	med/high	173
European Travel Skills	boring-1	3	low	low	119
Matcha: The way of Tea	boring-2	4	low	low	160
Relaxing Music with Beach	relaxing-1	5	med/high	low	145
Natural World: Zambezi	relaxing-2	6	med/high	low	147
Shutter	scary-1	7	low	high	197
Mama	scary-2	8	low	high	144
Great Barrier Reef	startVid	10	—	—	101
Blue screen with end credits	endVid	12	—	—	120
Blue screen	bluVid	11	—	—	120

### Sensors & instruments

The physiological sensors used for the experiment were selected based on their prevalence in AC datasets and applications^3,6,10,18^. Other sensors and instruments were chosen based on either the recommendations of the sensor manufacturer or on how interfaceable they were with the data acquisition setup. More details on these sensors and instruments are provided in this subsection and Table 2.

**Table 2:** The type, number (No.), manufacturer and model of different sensors and instruments used in the experiment.

Sensor/Instrument	No.	Manufacturer	Model	Conversions	
				Equations	Units
ECG sensor	1	Thought Technology	SA9306	${V}_{out}=({V}_{in}-2.8)/50\cdot 1{0}^{3}$	*mV*
BVP sensor	1	Thought Technology	SA9308M	$BVP{\rm{ \% }}=58.962{V}_{in}-115.09$	%
GSR sensor	1	Thought Technology	SA9309M	$G=24{V}_{in}-49.2$	*μS*
Respiration sensor	1	Thought Technology	SA9311M	$R{\rm{ \% }}=58.923{V}_{in}-115.01$	%
Skin temp. sensor	1	Thought Technology	SA9310M	$T=21.341{V}_{in}-32.085$	°*C*
EMG sensor	3	Thought Technology	SA9401M-50	${V}_{out}(RMS)=({V}_{in}-2)/4000\cdot 1{0}^{6}$	*μC*
Sensor Isolator	2	Thought Technology	SE9405AM	—	—
ADC module	1	National Instruments	NI 9205	—	—
DAQ system	1	National Instruments	cDAQ-9181	—	—
Joytick	1	Thrustmaster	T.16000M	$po{s}_{out}=0.5+9\cdot (po{s}_{in}+26225)/52450$	—

Wherever applicable, the conversion equations used to transform the logged input values to the desired output units/scales (see last column) are also presented.

#### ECG sensor

The electrical signal generated by the heart muscles during contraction can be detected using an ECG sensor. The used procedure involves placement of three electrodes in a triangular configuration on the chest of the participant. First, the skin placement site was prepared by (i) if required, removing any excess hair around the site, (ii) abrading the skin using the Nuprep abrasive cream, and (iii) cleaning the skin using an alcohol (70% isopropanol) pad. Then, pre-gelled electrodes were placed on the cleaned sites, such that two electrodes rest on the right and left *coracoid processes* and the third on the *xiphoid process*^38^. This sensor also pre-amplifies and filters the detected electric signal.

#### Respiration sensor

The expansion and contraction of the chest cavity can be measured using a Hall effect sensor placed around the *pectoralis major* muscle^38^. Accordingly, a respiration sensor belt was placed high on the torso (i.e., under the armpits but above the breasts). The tautness of sensor belt was set such that it was comfortable for the participants^38^. Since this sensor was worn over clothing, no skin preparation was required.

#### BVP sensor

Also known as a Photoplethysmography (PPG) sensor, it emits light into the tissue and measures the reflected light. The amount of observed reflected light varies according to the blood flowing through the vessels, thus serving as a measure for cardiac activity. A hand-based BVP sensor was used for this experiment and was placed on the middle finger of the non-dominant hand^38^. The use of this sensor does not mandate any specific skin preparation. However, to prevent any impediment in the functioning of this sensor resulting from dirt on the skin, the participants were asked to wash their hands with water. Following which, the sensor was placed and secured using an elastic band.

#### GSR sensor

Also known as Electrodermal Activity (EDA) sensor, it measures the variation in electrical conductance resulting from sweat released by the glands on the skin. Since these glands are regulated by the sympathetic nervous system, changes in electric conductance serve as a good indicator of physiological arousal. This sensor was placed on the index and ring fingers of the non-dominant hand^38^. The placement procedure followed for the same, involved: (i) rinsing the skin sites by water to remove any dirt followed by a pat-drying, (ii) applying conductance paste (Ten20^®^ that has 12.5% NaCl content) to the sites, and (iii) attaching the silver cloride (Ag-AgCl) electrodes in a bipolar configuration to the sites using finger straps. The hydration-level and the electrolytic concentration of the skin influence the level of electrodermal activity. Thus, a waiting-period of 5 to 15 minutes was allowed to stabilize the skin-electrolyte interface. During this waiting-period, other sensors were placed and the sensitivity of this sensor was verified by asking the participants to take a deep breath and hold it for few seconds. A good GSR signal showed a spike in skin conductance within a couple of seconds of the breath being initiated^39^.

#### Skin temperature sensor

Small variations in skin temperature were measured and converted to electrical signals using an epoxy rod thermistor. This sensor was placed on the pinky finger of the non-dominant hand^38^, where it was secured using Coban self-adhesive tape that allowed for a snug placement.

#### EMG sensors

The surface voltage associated with muscle contractions can be measured using a surface-Electromyography (sEMG, simply EMG) sensor. Previous research in AC has generally focused on three muscles. These are the *zygomaticus major* and the *corrugator supercilii* muscle groups on the face, and the *trapezius* muscle on the upper-back. Accordingly, a total of three EMG sensors (one each for the aforementioned muscles) were used for this experiment. The skin at the placement sites for these sensors was prepared by gently abrading it using the Nuprep cream. The details on the placement of the pre-gelled electrodes for these sensors are as follows:

1. *zygomaticus major* – The first electrode was fixed midway along an imaginary line joining the *cheilion* and the *preauricular* depression, i.e., at the bony dimple above the posterior edge of the *zygomatic arch*. The second electrode was placed 1 cm inferior and medial to the first, i.e., approximately at the point where the horizontal *sub-nasale* line first intersects the *zygomaticus major* muscle. The third electrode serves as a reference electrode and was placed at the left/right upper edge of the forehead, i.e., ~1 cm below the hairline.
2. *corrugator supercilii* – The first electrode (surface area: ~6 cm^2^) was fixed above the brow on an imaginary vertical line that extends over the *endocanthion* (i.e., the point at which the inner ends of the upper and lower eyelid meet). The second electrode was placed 2 cm to the right and slightly superior the first electrode such that it sits at the upper edge of the eyebrow. The third electrode serves as a reference electrode and was placed at the central upper end of the forehead, i.e., ~2 cm below the hairline.
3. *trapezius* – A triode electrode (surface area: ~30 cm^2^) was placed at the edge between superior and middle fibres of the *trapezius* muscle, approximately 10 cm left/right from the first *thoracic vertebra* for a 180 cm tall adult.

After the placement of each sensor’s electrodes, the impedance between them was checked and verified to be within the range specified by the sensor manufacturer (i.e., 0–15 kΩ for a reliable assessment). Following this evaluation, the sensor leads from the main sensor unit were connected to the electrodes. The main sensor unit pre-amplifies and filters the acquired raw EMG signal, and also perform an analog Root Mean Square (RMS) conversion of the same^38^.

#### Sensor isolators

**Fig. 3:**
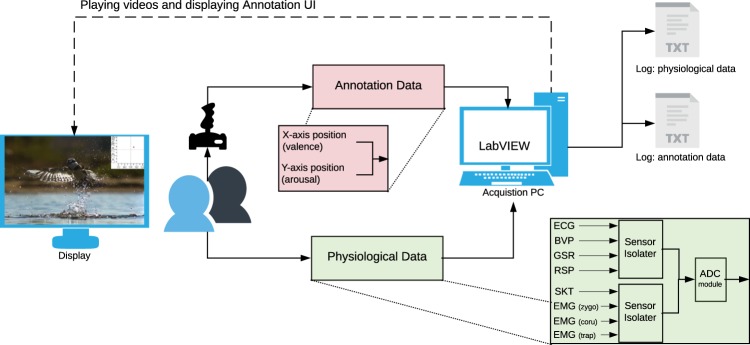
The schematic shows the various aspects of the experiment and the data acquisition setup. The arrows indicate the direction of the data-flow. The solid and the dotted lines indicate the primary and secondary tasks of the acquisition process, respectively.

The sensor manufacturer recommends using a ‘sensor isolator’ to ensure electrical isolation between the participants and the powered sensors. Accordingly, the physiological sensors were indirectly connected to the data acquisition module, through these sensor isolators (see Fig. 3).

#### Data acquisition modules

A 32-channel (16-channel differential) Analog-to-Digital Conversion (ADC) module with 16-bit resolution was used to acquire the output voltages from the sensor isolators (indirectly, the sensors). This module was connected to a Data Acquisition (DAQ) system that transfers the data to the acquisition PC.

#### Joystick

The joystick was the only instrument in the experiment that is directly controlled by the participants. The used joystick is a generic digital gaming peripheral that features a return spring. This provided the user proprioceptive feedback about the location of the pointer in the interface, thereby helping to mitigate the cognitive load associated with simultaneous tasks of watching the video and annotating the emotional experience^16,25^.

### Data acquisition

Figure 3 shows the experiment and the data acquisition setup. The video-playback, the annotation interface and the data acquisition components were all directly managed through LabVIEW. This allowed for a seamless integration of all these different components. The open-source VLC media player was used for video-playback. The joystick was directly connected to the acquisition PC over a USB port. The physiological data was acquired over Ethernet using the DAQ system. The acquisition rate for the annotation and the physiological data was 20 Hz and 1000 Hz, respectively. The acquired data was augmented with the timestamp provided by the acquisition PC and logged in two different text files, i.e., one each for the physiological and the annotation data. The same process was repeated for all participants resulting in 60 (30 × 2) log files.

### Data preprocessing

The procedure used for processing the raw log files is summarized in this subsection. In the following, step 1 was performed once and steps 2–6 were iteratively applied to log files for each participant.

1. *Duration of the videos:* using the *ffprobe* tool from the *FFmpeg* multimedia framework^40^, the exact duration (in milliseconds) of the videos was determined and has been made available in the dataset^31,32^.
2. *Initial files:* the raw log files for the physiological data were generally large in size (~200 MB) and hence manipulating these files in MATLAB was very slow. To this end, the raw data from both, the annotation and physiological files, was first extracted and then saved in a single MATLAB preferred*.mat* format file. The subsequent steps in preprocessing were implemented on the data stored in these *mat* files.
3. *Transforming raw data:* the sensor input received by the sensor isolators gets modified before being transferred to the DAQ system. To rectify the effects of this modification, the logged voltages need to be transformed. This was achieved by applying the equations presented in Table 2 to yield the desired output with specific units/scales (Table 2, last column). Similarly, the logged annotation data, which was in the integer interval $[-26225\,..26225]$, was also rescaled to the annotation interface interval $[0.5\,..9.5]$ by using the equations presented in Table 2.
4. *Data interpolation:* a common problem in data acquisition and logging is the latencies that can be introduced during any of these processes. This was also evident in our data, where, e.g., the time between the subsequent samples of the annotation data was occasionally more than the expected 50 ms. To address this issue, linear interpolation was performed on the physiological and the annotation data. For undertaking the same, first, two time-vectors with sampling intervals of 1 ms (for the physiological data) and 50 ms (for the annotation data) were generated based on the time-duration of the logged data. These vectors serve as the query points for the interpolater that determines the value at these points by fitting a line between the corresponding discrete samples in the logged data. As a result of the interpolation process, the resulting sampling intervals for the physiological and the annotation data were 1 ms and 50 ms, respectively. In the case that other researchers might prefer to use either the non-interpolated data or different interpolation methods, the original non-interpolated data are also available in the dataset^31,32^.
5. *Adding the video-IDs:* the log files contain timestamps, but do not have information identifying the duration and the order of the videos. Hence, the extracted video-durations and a lookup table of the video-sequences were used to identify the data segments pertaining to each video. Then, this information was added as an extra column in the log files, containing the different video-IDs (see Table 1). This process was also undertaken for the non-interpolated data.
6. *Saving the data:* the resulting data from the aforementioned steps was saved into two different *comma-separated values* (csv) files, i.e., one each for the physiological and the annotation data. The csv format was chosen as it is natively accessible by different programming and scientific computing frameworks.

## Data Records

The presented CASE dataset is available in three variants:

- **CASE_full**: includes raw, initial and processed data from all 30 participants, and also the preprocessing code and other metadata. This version of the dataset is the most comprehensive and should ideally be the first choice for users interested in performing downstream analyses on the data. This dataset is hosted as a single archive file on the *figshare* data repository^31^.
- **CASE_snippet**: includes raw, initial and processed data from only 2 participants, and also the preprocessing code and other metadata. The size of **CASE_full** archive is ∼5 gigabytes (GB). Hence, to allow users to examine CASE before downloading the full dataset, we created this snippet of the dataset. The size of the snippet archive file is only ~0.3 GB and it is also hosted on the *figshare* data repository^31^.
- **CASE git repository**: includes raw data from all 30 participants, the preprocessing code and other metadata. This repository offers the users yet another convenient method to examine CASE and its preprocessing code. Users can hence easily browse through the preprocessing code on the repository website and if desired, clone the repository and reproduce the processed data. Users can also verify the processing pipeline by comparing the reproduced processed data with the processed data available in **CASE_full**. This *git* repository is hosted on *GitLab*^32^ and more information on it is available in the Usage Notes section.
  The directory structure implemented across the aforementioned versions of CASE dataset is the same. At the root of the dataset, it is organised into following three main directories: (i) *data*, (ii) *metadata* and (iii) *scripts*. Detailed *README* files that explain the contents of each directory and any subsequent sub-directories are available in these directories.
  Some pointers that are essential to understand this section and the dataset in general, are as follows:
- The following description of the data records uses the letters XX to denote the IDs of the participants, where XX are natural numbers in the set {1, 2, …, 30}. Thus, a combination of XX with a specific filename (e.g., sub_XX.csv) is used to denote that the file exists for all 30 participants.
- The *jstime* and the *daqtime* variables mentioned in the following subsections contain timestamps provided by a common clock on the logging computer. They have been named and stored separately due to the different sampling intervals used for logging of the annotation and physiological data, i.e., 50 ms and 1 ms, respectively.

### Data

The *data* directory is further divided into the following sub-directories: (i) *raw*, (ii) *initial*, (iii) *interpolated* and (iv) *non-interpolated*. These sub-directories and the data contained in them pertain to the different stages of the data acquisition and preprocessing pipeline.

### Raw

This directory contains the raw data logs acquired using LabVIEW (see Data Acquisition). It is further divided into: (i) the *annotations* and (ii) the *physiological* sub-directories that contain the participant-wise annotation and physiological data, respectively. An overview of the data files in these sub-directories is provided below.

**Annotations/**subXX_joystick.txt – contains 30 raw annotation data files titled subXX_joystick.txt. Each file contains the following three variables (1 variable per column):

- *Column 1*. Time in seconds from the beginning of the video-viewing session to the end.
- *Column 2*. The X-axis value (i.e., valence) of the joystick position in the interface. The values lie in the integer range from −26225 to +26225.
- *Column 3*. The Y-axis value (i.e., arousal) of the joystick position in the interface. The values lie in the integer range from −26225 to +26225.
  **Physiological/**subXX_DAQ.txt – contains 30 raw physiological data files titled subXX_DAQ.txt. Each file contain the following nine variables (1 variable per column):
- *Column 1*. Time in seconds from the beginning of the video-viewing session to the end.
- *Columns 2–9*. Contain the input voltages (measured in volts) from the ECG, BVP, GSR, Respiration, Skin temperature, EMG-*zygomaticus*, EMG-*corrugator* and EMG-*trapezius* sensors, respectively.

### Initial

As previously mentioned in Data Preprocessing, the raw physiological and annotation data from each participant was loaded into MATLAB and subsequently saved into a participant-wise *mat* file (e.g., sub_XX.mat). The *mat* files for all 30 participants are stored in the *initial* directory. Each *mat* file contains 12 variables. Three of these variables, i.e., *jstime* (joystick time), *val* (valence) and *aro* (arousal), pertain to the raw annotation data. The rest, i.e., *daqtime* (DAQ time), *ecg*, *bvp*, *gsr*, *rsp* (respiration), *skt* (skin temperature), *emg_zygo* (EMG-*zygomaticus*), *emg_coru* (EMG-*corrugator*) and *emg_trap* (EMG-*trapezius*), pertain to the raw physiological data. The data contained in these *mat* files is the same as the data in the *raw* directory. Nevertheless, it was added to the dataset to offer a convenient starting point for MATLAB users.

### Interpolated and non-interpolated

These directories contain data that results from steps 3–5 mentioned in the Data Preprocessing section. They are structured like the *raw* directory and hence are further divided into the: (i) *annotations* and (ii) *physiological* sub-directories. The only difference between the data contained in these directories is that the generation of *interpolated* data involves an extra processing step (see Data Preprocessing). Hence, unless stated otherwise, the description of the data records provided below is applicable to the files in both of these directories.

**Annotations/**sub_XX.csv – the annotation data files (titled sub_XX.csv) from all 30 participants are contained in this directory. The names (variable-names) and the contents of the four columns in each file, are as follows:

- *Column 1: jstime*. Time in milliseconds from the beginning of the video-viewing session to the end.
- *Column 2: valence*. The scaled X-axis value of the joystick position in the interface (see Table 2).
- *Column 3: arousal*. The scaled Y-axis value of the joystick position in the interface (see Table 2).
- *Column 4: video*. Contains the sequence of video-IDs that indicates the ordering and duration of the different video-stimuli for the given participant.
  **Physiological/**sub_XX.csv–contains the physiological data files (titled sub_XX.csv) for all 30 participants. The names (variable-names) and the contents of the 10 columns in each file, are as follows:
- *Column 1: daqtime*. Time in milliseconds from the beginning of the video-viewing session to the end.
- *Columns 2–9: ecg, bvp, gsr, rsp, skt, emg_zygo, emg_coru* and *emg_trap*. The transformed sensor output values for each of 8 physiological sensors used in the experiment. More information on the sensors, the transformations applied, and the outputs units for these values, is available in Table 2 and the README files in these directories.
- *Column 10: video*. Contains the sequence of video-IDs that indicates the ordering and duration of the different video-stimuli for the given participant.

### Metadata

This directory contains auxiliary information about the experiment that has been organised into the following files:

- participants.xlsx – this Excel file contains the participant-ID, sex, age-group and ID of the video-sequence used, for all participants in the experiment.
- seqs_order.txt, seqs_order_num.mat and seqs_order_num.xlsx – the video-stimuli were shown in a unique sequence to every participant. The columns of these files contain either the video-labels (in seqs_order.txt) or the video-IDs (otherwise) that indicate the ordering of the video-stimuli in these sequences. The seqs_order_num.mat is created during preprocessing from seqs_order.txt. The data in seqs_order_num.mat and seqs_order_num.xlsx is the same and the latter has been added to the dataset only for the convenience of users.
- videos_duration.txt, videos_duration_num.mat and videos_duration_num.xlsx – contain the duration in milliseconds of the different video-stimuli used for this dataset. As was the case in the previous point, these files contain the same information only in different formats. Where, video-labels are used in videos_duration.txt and video-IDs otherwise. Similarly, videos_duration_num.xlsx has been included in the dataset only for the convenience of users.
- videos.xlsx – in addition to the attributes already presented in Table 1, this Excel file contains further information on the used video-stimuli. This includes, the videos’ durations in milliseconds, links to the IMDb/YouTube entries for the videos’ sources, URLs to the videos and the time-window for the videos at these URLs. More information on how to acquire these videos is presented in the Usage Notes section.

### Scripts

This directory contains the code (MATLAB scripts) used for preprocessing the acquired raw data. Since preprocessing is a multi-step process, the code has been appropriately divided across the following scripts:

1. s01_extractVidData.m – the duration information acquired from step 1 of Data Preprocessing is saved in videos_duration.txt. Similarly, seqs_order.txt contains the sequence of videos for all participants. This script extracts the data from these *txt* files, converts the video-labels to video-IDs and saves the converted data to *mat* files.
2. s02_extractSubData.m – implements the preprocessing measures mentioned in the second step of Data Preprocessing. The resulting files are saved in the *initial* data directory.
3. s03_v1_transformData.m – is used to generate the *non-interpolated* data. It implements steps 3, 5 and 6 of Data Preprocessing, in that order.
4. s03_v2_interTransformData.m – is used to generate the *interpolated* data. It first inter-/extra-polates the raw data to standardize the sampling intervals (see step 4 of Data Preprocessing). Then, in a similar fashion to s03_v1_transformData.m, steps 3, 5 and 6 of Data Preprocessing are implemented.
5. f_labelData.m – implements step 5 of Data Preprocessing. This script is used as a helper function by scripts s03_v1_transformData.m and s03_v2_interTransformData.m to label the transformed data.

## Technical Validation

### Annotation data

The quality and the reliability of the annotation data has been thoroughly addressed in our previous works^16,27–29^. A summary of the relevant highlights from these works is presented below.

In^27,28^ several different exploratory data analyses were presented. These analyses provided an initial intuition into the annotation patterns for the different video-stimuli. For example, the annotations for the two scary videos had in general low valence and high arousal values. They were thus different from annotations for the amusing videos which had relatively high valence and medium arousal. These differences can also be seen in the annotations presented in Fig. 2 (left). The initial exploratory results presented in^27,28^ were then formally validated in^16^, where Multivariate ANOVA (MANOVA) was used to quantify the statistical significance of the differences in the annotations for these videos. The ‘usability’ of our annotation approach was validated using the *System Usability Scale* (SUS) questionnaire. According to the ratings received on the same, the annotation setup had ‘excellent’ usability as the participants found it to be consistent, intuitive and simple to use^16^. In^16,29^, several different methods for analysing the annotation patterns in continuous 2D annotations were presented. The results of these continuous methods were concurrent to the results of the MANOVA. Also in^16,29^ several different methods for extracting additional information from these continuous annotations have been presented. For example, the *Change Point Analysis* method in^16^ automatically detects the major change-points in the annotation data that can be used to segment the annotations into several salient segments. For comparison with the physiological data, some results for the annotation data are presented in the next subsection.

### Physiological data

In the Background & Summary section, the typical AC processing pipeline was presented. The final objective of this pipeline is to develop machine learning models that can infer the emotional state of humans from (in the given case) physiological signals. To achieve the same, it is critical that the physiological responses to the different video-stimuli are discernible from each other and are ideally correlated to annotation data. If indeed these patterns exist, they would validate the quality and the value of this data. To determine the same, we extracted several features from the *interpolated* physiological data and performed *Principal Components Analysis* (PCA) on these features. The details and results of this analysis are presented as follows.

### Feature extraction

The feature extraction was performed iteratively over the physiological data files for each participant. First, the data for a given participant was segmented into chunks for the different video-stimuli. Then, from the sensor data pertaining to each of these video-chunks, several features were extracted (see Table 3).

**Table 3:** The sensors and the various features extracted from the sensor signals.

Sensor	Extracted Features
ECG	Heart Rate (HR)
	Inter-Beat Interval (IBI)
	Standard Deviation (SD) of NN-intervals (SDNN)
BVP	Heart Rate (HR)
	Inter-Beat Interval (IBI)
	Standard Deviation (SD) of NN-intervals (SDNN)
GSR	Skin Conductance Level (SCL)
	Skin Conductance Response (SCR)
Respiration	Respiration Rate (RR)
	Interval of Respiration peaks
Skin Temperature	Temperature
	SD of Temperature (SDT)
EMG–zygomaticus	Amplitude of the signal
EMG–corrugator	Amplitude of the signal
EMG–trapezius	Amplitude of the signal

**Fig. 4:**
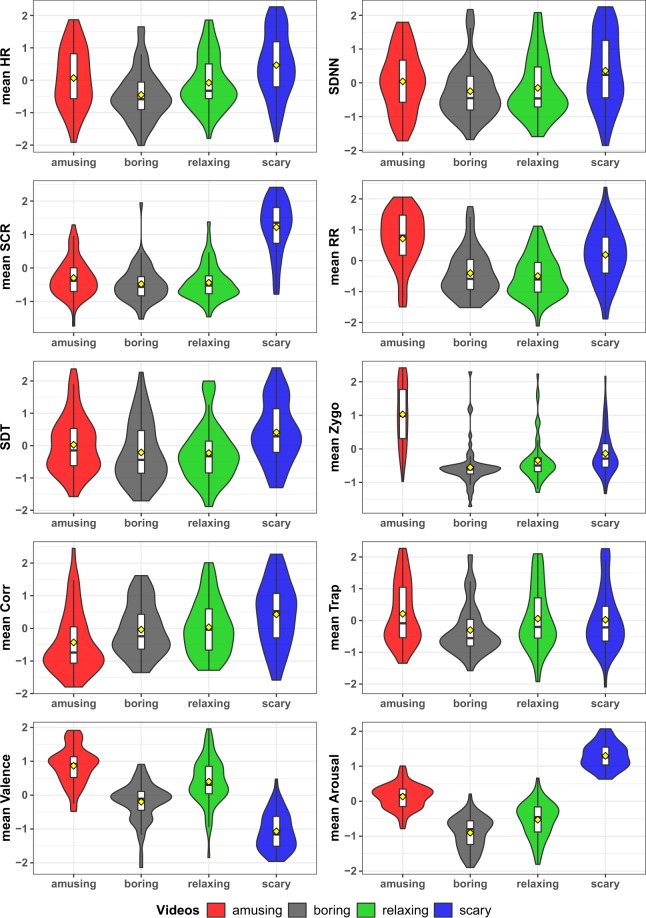
“Violin” plots of the distribution of the selected features and the mean annotation (valence & arousal) values across different types of videos. The box plots embedded in each violin plot show the Interquartile Range (IQR) for each considered variable, while a yellow diamond marks the mean of the distribution.

For the technical validation presented here, one predominantly used feature for each sensor was selected and where applicable, the mean of this feature across the given video-chunk was calculated. Similarly, the mean valence and arousal values across each video-chunk were calculated. The selected physiological features are presented in Table 4. As a result, for the 30 participants who all watched eight emotional video-stimuli, we have 240 (30 × 8) values for each of the selected features. Due to inter-personal differences, the participants have a different baseline value for each of these extracted features. These differences can be detrimental to the comparison of the features across all participants and were therefore removed using *Z-score* standardisation across each participant. The same was also done for the annotation data. The violin-plots in Fig. 4 show the distributions of the selected features and annotation data, across the 4 different video-labels (see Table 1). From the figure, it is apparent that some of the physiological features (consider the top eight panels) characterise specific types of videos. For instance, scary videos result in high values of SCR and elevated HR, while amusing videos elicit accelerated respiration rates and activity of the *zygomaticus* muscles. Boring and relaxing videos, as expected, elicit similar values of all features. These results are in line with previous research^5,6^, where, e.g., HR and SCR were determined to be positively correlated to arousal. This effect can also been seen in our data, where the reported arousal levels (see bottom-left panel) for scary videos are higher than for the other videos. Similarly, *zygomaticus* activity which has been reported to be positively correlated to valence (see bottom-left panel), also exhibits similar patterns in our data.

**Table 4:** The sensors and the features selected from each sensor.

Sensor	Feature Selected
ECG	mean HR
BVP	Standard Deviation (SD) of NN-intervals (SDNN)
GSR	mean SCR
Respiration	mean RR
Skin Temperature	SD of Temperature (SDT)
EMG–zygomaticus	mean amplitude (mean Zygo)
EMG–corrugator	mean amplitude (mean Corr)
EMG–trapezius	mean amplitude (mean Trap)

### PCA

**Fig. 5:**
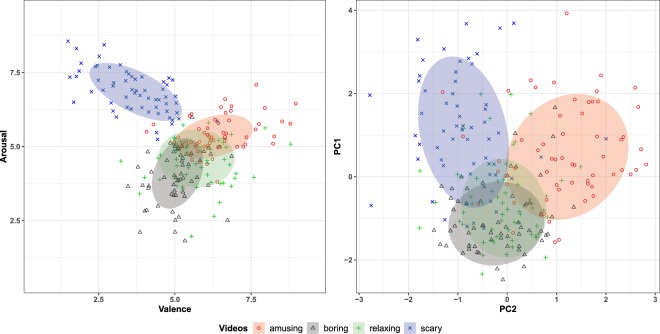
Scatter plots of the mean annotation data and the first two principal components of the physiological data, labelled according to the types of videos. Ellipses denote one standard deviation.

PCA is a commonly used dimensionality reduction technique^41,42^. Thus, it allows for visualization of the given data in a lower dimensional space, where e.g., spatial distributions of the data can be analysed. To this end, PCA was undertaken on the selected *Z-scored* features listed in Table 4. For the analysis presented here, that compares the distribution of the PCA scores for these features to the 2D annotation data (see Fig. 5), only the first two Principal Components (PC) were retained. These PC explain 43% of the variance in the selected features (1.PC: 27% and 2.PC: 16%). The scatter plot on the left in Fig. 5 shows the mean valence and arousal values across the different video-labels. The data ellipses show the standard deviation of data pertaining to these video-labels. As is evident from this figure, the physiological and the annotation data form concurrent clusters. These two figures validate the data in an even more prominent way than Fig. 4. Valence and arousal values (left panel) of scary videos are concentrated in the upper-left quadrant, those for the amusing videos are in the upper-right, and the others lie in the middle with low arousal values, as one would expect. This is confirmed by the right panel, in which the four types of videos are represented analogously on the plane obtained using the first two principal components of the physiological features. This seems to indicate that the physiological features somehow “match” the joystick annotations. Of course, this serves only an initial investigation and a more rigorous analysis is required to fully exploit the potential of the database. Nevertheless, the results provided here show that the presented dataset has several viable characteristics that would make it of interest to our research community.

## Usage Notes

### Videos

Due to copyright issues, we cannot directly share the videos as a part of this dataset^31,32^. Nonetheless, to help users in ascertaining the emotional content of these videos in more detail and if required, to replicate the experiment, we have provided links to websites where these videos are currently hosted (see /metadata/videos.xlsx). We are aware that these links might become unusable in the future and that this can cause inconvenience to the users. In such an eventuality, we encourage the users to contact us, so that we can assist them in acquiring/editing the videos.

### GitLab repository

The code contained in this repository^32^ will not be updated such that it remains identical to the code available in **CASE_full** and **CASE_snippet**. To ensure the same, as an extra measure, we have created a ‘release’ for this repository. This release points to a snapshot of this repository that is concurrent to the compilation of **CASE_full** and **CASE_snippet**. This release is available at: https://gitlab.com/karan-shr/case_dataset/tree/ver_SciData_0.

Also, users who require assistance with the dataset are welcome to contact us using the ‘issues’ feature from *GitLab*.

### Feature extraction and downstream analysis

The code used for the technical validation of the dataset was developed in MATLAB 2014b and R-language (version 3.3.3). The feature extraction was done in MATLAB using open-source toolboxes/code like TEAP^43^ and an implementation of the Pan Tompkins QRS detector^44^. The PCA analysis was performed in R using the *prcomp* function from the *stats* package. This code is available to interested researchers upon request. Users of the dataset^31,32^ interested in leveraging the continuous nature of the provided annotations are advised to check our previous works^16,29^. The analysis presented in these works was primarily undertaken in R-language and can be easily reproduced.

## Supplementary Information

Supplementary file

## Data Availability

The LabVIEW-based graphical code for the experiment and data acquisition is highly specific to the sensors and equipment used in our experiment. It has therefore not been made available with this dataset^31,32^. Nevertheless, readers who wish to replicate the experiment can contact the corresponding author for further assistance. For readers who want to reproduce the experiment, we hope the detailed description provided in this article will suffice. The data preprocessing steps outlined in the previous subsection were implemented in MATLAB 2014b. The linear interpolation was performed using the *interp1* function. The raw log files and the data preprocessing code are available in the dataset^31,32^. Hence, readers who wish to reproduce and/or improve-upon our preprocessing pipeline can easily undertake the same.
