# Supplementary material for: A dataset of continuous affect annotations and physiological signals for emotion analysis
Source: Sci Data. 2019 Oct 9;6:196. doi: 10.1038/s41597-019-0209-0 (PMC6785543; doi:10.1038/s41597-019-0209-0)
Supplement: Supplementary file 1 — Supplementary file [file 41597_2019_209_MOESM1_ESM.pdf]

Information on the Test

This document is divided into two parts:

1. User Feedback UI (Joystick based User Interface).
2. Information on Sensors.

Please read the first part of the document. It will help you to grasp the idea behind the User Interface. **You are not required to read the second part of the document;** it's there only for those who are interested in knowing more about the sensors.

User Feedback UI:

While watching the videos, you will be required to give feedback on your emotional states. Since we don't want to interrupt you during the video, we have designed a joystick based feedback system that allows you to express your emotional state by moving the joystick into different regions of the user interface. By the means of this interface, you can answer the following questions:

1.) How pleasant do you feel while watching the video clip?

2.) How aroused/excited do you feel while watching the video clip?

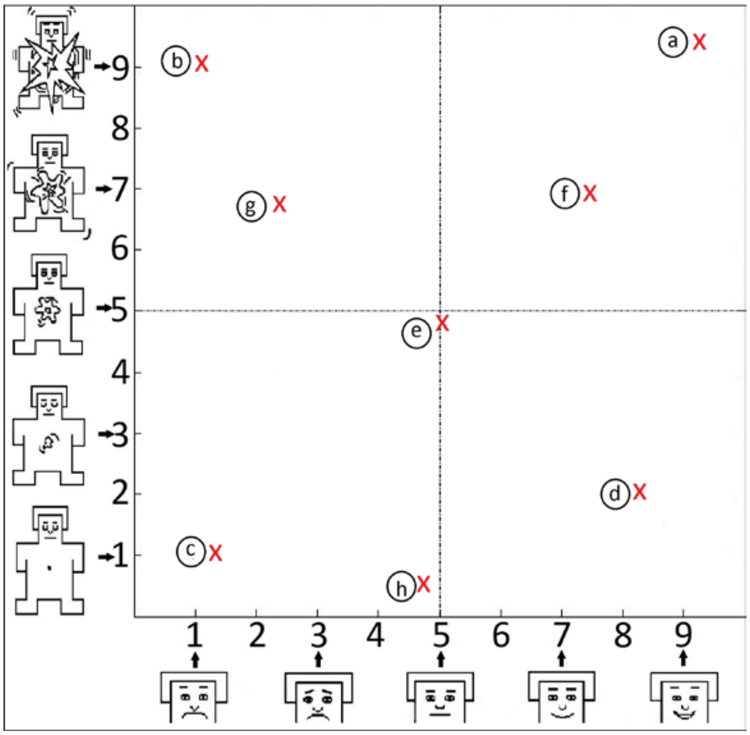

The figure above shows a screenshot of the user feedback UI. The horizontal axis ranges from 1 to 9, with 1 representing ‘displeasure’ and 9 depicting a ‘highly pleasurable’ state. Similarly, the vertical axis ranges also from 1 to 9, with 1 being low arousal (the human figure has his eyes shut) and 9 depicting ‘high arousal’ (the figure is visibly very excited).

While watch the videos, the UI will be visible on the upper left hand corner of the screen. During the video, you should position the joystick at that location that best describes your feeling at that given time. For example, let’s look at a few joystick positions (marked on the figure) that you might want to move to during the test:

a.) This position on the figure means: You are highly excited, as well as happy. Similarly,

b.) You are highly excited, but the feeling is very unpleasant,

c.) The feeling is highly unpleasant as well as least exciting,

d.) You are happy and calm,

e.) You are neither excited nor calm. Also, neither feeling pleasant nor unpleasant,

f.) Intermediate levels of pleasantness and excitement,

g.) Medium displeasure and medium excitement,

h.) Excitement levels are low, and neither pleasant nor unpleasant.

The above illustrations are provided to introduce you to the User Feedback UI. Even though it seems overly complex, it’s not! We’ve tried this UI with a couple of users before and based on their feedback, one gets the hang of this system after some training. That’s why on the day of the test, we’ll have a 15 minute training session with this system before the main test. **So, do not worry or feel stressed if you didn’t understand the system.** We want your participation to be as pleasurable as possible.

<u>Information on Sensors:</u>

These sensors will be worn prior to the experiment. A short description of the sensors and their positions is presented below:

1.) <u>ECG sensor:</u> It measures the heart rate. An ECG sensor has 3 electrodes and it is placed in a triangular configuration on the body, as illustrated below:

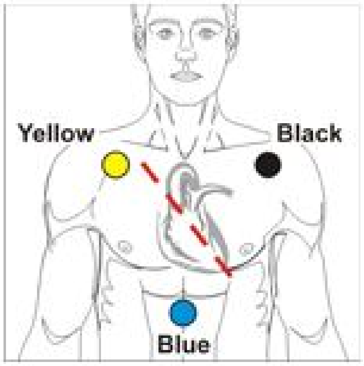

2.) Skin conductance sensor: It measures the skin potential (EDA-Electrodermal Activity). The index and ring finger of the left hand are used for the attachment of the sensors.

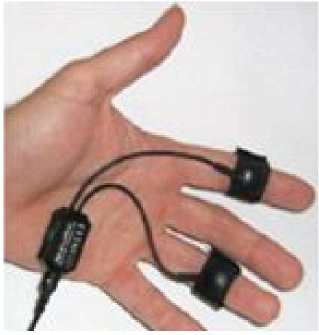

3.) EMG sensors: They are used for measuring the muscular activity of the facial and shoulder muscles. The EMG sensors are placed on the face and the shoulder, as shown below:

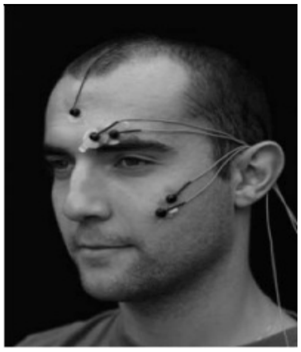

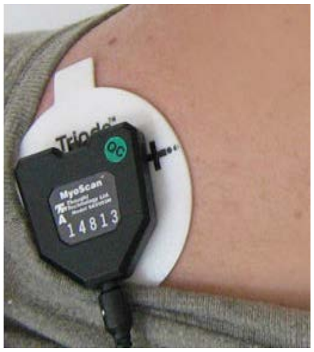

4.) BVP sensor: It measures the blood flow volume. It is attached to the middle finger of the left hand as shown.

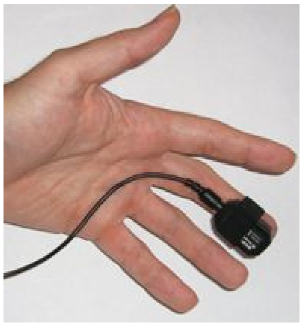

5.) Respiration sensor: measures the respiration rate. It is worn on the chest as shown.

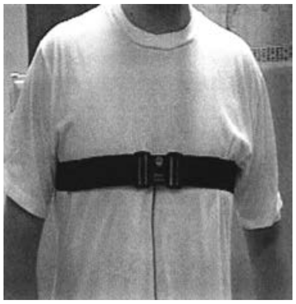

6.) <u>Temperature sensor:</u> measures the body temperature. It is attached on the ring finger as shown.

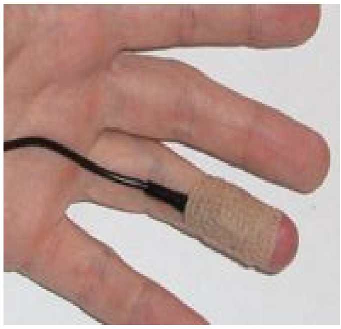

This document presented a brief overview of the User Feedback UI and the sensors. **Again, please don't stress yourself if you are not able to follow the topics entirely**. We want your participation to be as pleasurable as possible. Thank you once again for agreeing to take part in the test. We are very grateful for your effort.

Best Regards,
Karan
